# Supplementary material for: The synergistic effects of microcredit access and agricultural technology adoption on maize farmer’s income in Kenya
Source: PLoS One. 2025 Jan 6;20(1):e0316014. doi: 10.1371/journal.pone.0316014 (PMC11703112; doi:10.1371/journal.pone.0316014)
Supplement: S1 Table — (DOCX) [file pone.0316014.s001.docx]

**Supporting information**

**Appendix.**

**S1 Table. Test for Multicollinearity and heteroscedasticity**

| Variables | VIF | 1/VIF |
| --- | --- | --- |
| Cooperative Membership | 1.83 | 0.54 |
| Farming Experiences | 1.60 | 0.62 |
| Sources of information | 1.44 | 0.69 |
| Land size | 1.23 | 0.81 |
| Fertilizer use by households | 1.16 | 0.86 |
| Educational level in years | 1.15 | 0.87 |
| Number of livestock in household | 1.13 | 0.88 |
| Marital Status | 1.06 | 0.94 |
| Off-farm employment | 1.04 | 0.96 |
| Age | 1.03 | 0.96 |
| Mean VIF | 1.27 |  |
| Heteroscedasticity |  |  |
| Test | X2 | P value |
| Breusch-Pagan (BP) test | 0.025 | 0.97 |

Source: Source: Author's survey
