# Supplementary material for: The synergistic effects of microcredit access and agricultural technology adoption on maize farmer’s income in Kenya
Source: PLoS One. 2025 Jan 6;20(1):e0316014. doi: 10.1371/journal.pone.0316014 (PMC11703112; doi:10.1371/journal.pone.0316014)
Supplement: S1 File — (DOCX) [file pone.0316014.s002.docx]

SURVEY DATA

**1. what is your age?**

18-24 years

25-34 years

35-44 years

45-54 years

55-64 years

65 years and above

**2. Gender**

Female

Male

**3. what is your marital status?**

Married

Single

Divorced

widowed

separated

**4. What is your highest education level?**

Primary

Secondary

Tertiary

None

**5. Household size?**

1-5

5 and more

**6. How long have you been practicing maize farming (Experience**)

Less than 1 year

1-2 years

3-5 years

6-10 years

more than 10 years

**7. What is your major occupation………….?**

Farming

Employed

Business Activities

other

Land utilization and Preparations

Description (optional)

**8.a) What is the total land size in your farm___________(acres)?**

Less than 1 acre

1-5 acres

6-10 acres

11-20 acres

More than 20 acres

**8b. What size of your land was used for maize production in the 2023/2024 financial year**?

Less than 1 acre

1-5 acres

6-10 acres

11-20 acres

More than 20 acres

**Not applicable/ I did not plant this**

**8c) Whether Household received Extension education from 3-6 months during the maize plantation period**

Yes

No

**8d) What is the mode of acquisition of your land?**

1 = Inherited

2 = Bought

3 = Tenancy

4 = Borrowed

5 = Others (Specify): __________

**8e), If tenant, what type of tenancy arrangement did you operate? Tick appropriately.**

Fixed rent

Share-cropping

**8f) If fixed rent, please specify the duration of tenure:**

Less than 1 year

1-5 years

6-10 years

More than 10 years Option 1

Credit Access Information

Description (optional)

**9a). Do you have a bank Account?**

Yes

No

**9b). Do you require credit/loans to purchase the improved maize varieties and chemical fertilizer?**

Yes

No

**9c) Do you access credit for farming activities?**

Yes

No

**9d), If yes, please specify the source(s) of credit**

Commercial banks

Agricultural Finance Cooperation’s. (AFC)

NGO/Non-profit organization

Family or friends

Private organizations, One Acre Fund /Apollo Agriculture

Option 7

**9e). What purposes do you typically use credit for in your maize farming activities?**

Purchase of seeds

Fertilizer and agrochemicals

Equipment and machinery

**Other (please specify) same**

**10a). Which of the following factors influences the farmer decision most during the loan application process**?

Interest rates

Loans Terms and Conditions

Collateral Requirements

Credit History

Lender Reputations

Loan Amount required by farmers

Accessibility and Convenience of financial Institutions

**10b). Have you ever faced any challenges in repaying the credit obtained for maize farming**?

Yes

No

**10c). If yes, please select the challenges you encountered in repaying the credit.(Select all that apply)**

Loan Terms and Interest Rates

Pest and Disease Outbreaks

Yield Variability

Market Price Fluctuations

Personal Health Issues

Economic and Livelihood Impact:

**Credit general Impact**

**10d). How has maize farming, with the help of credit, contributed to your household income?**

significant increase

Moderate increase

No significant change

Decrease

Not sure

**10e). Have you been able to save or invest in other income-generating activities as a result of maize farming with credit?**

Yes

No

**10f). Do you anticipate needing credit for maize farming in the upcoming planting season?**

Yes

No

**10g). What is your level of satisfaction with credit access offered by financial providers?**

Satisfied

Not Satisfied

Average

Undecided

**10h). What measures/policies, if any, do you think would address the cycle dependency of credit by farmers (select all that apply):**

Diversification of Income Sources

Training and Capacity Building

Access to Savings and Insurance

Access to Technology

Access to Markets

Value Addition and Agro-Processing

Improved Maize Varieties Adoption

**Description (optional)**

**11a) Have you adopted any improved maize varieties in the last three years?**

1. Yes [ ] 2.No

**11b). If yes, please list the types of improved maize varieties you have adopted.**

H614

H613

H6213

H629

**Others**

**11c). What factors influenced your decision to adopt improved maize varieties?**

Higher yield

Disease resistance

Drought tolerance

Other (please specify)

**11d). How much Quantity of Improved Maize Seeds were used?**

Less than 5 kilograms

5-10 kilograms

11-20 kilograms

21-50 kilograms

More than 50 kilograms

Not sure/Don't remember

**11e), Did you receive any subsidized farm inputs during last purchasing period?**

Yes

No

**12a. How much Quantity of Fertilizer used in Kgs?**

Less than 10 kg

10-20 kg

21-50 kg

51-100 kg

More than 100 kg

**Not sure/Don't remember Option 1**

**12b) Whether you were influenced by social interactions / Observation from others to adopt improved maize varieties**

Yes

No

**12c). Over the past 12 months (Jan 2023-Dec 2023), how much money (Ksh) did you spend on the following inputs and services on IMPROVED maize?**

**COST OF PRODUCTION**

a**) Land Preparation**

Less than 1,000 Ksh

1,001-5,000 Ksh

5,001-10,000 Ksh

10,001 -20000ksh

20,001-40,0000

40,001-60,000ksh

60001-80000ksh

80001-100000ksh

More than 100,000ksh

**b) Plantations**

Less than 1,000 Ksh

1,001-5,000 Ksh

5,001-10,000 Ksh

10,001 -20000ksh

20,001-40,0000

40,001-60,000ksh

60001-80000ksh

80001-100000ksh

More than 100,000ksh

c**) Weeds Control**

Less than 1,000 Ksh

1,001-5,000 Ksh

5,001-10,000 Ksh

10,001 -20000ksh

20,001-40,0000

40,001-60,000ksh

60001-80000ksh

80001-100000ksh

More than 100,000ksh

**d) Pesticides cost entire period**

Less than 1,000 Ksh

1,001-5,000 Ksh

5,001-10,000 Ksh

10,001 -20000ksh

20,001-40,0000

40,001-60,000ksh

60001-80000ksh

80001-100000ksh

More than 100,000ksh

**e) Top dressing**

Less than 1,000 Ksh

1,001-5,000 Ksh

5,001-10,000 Ksh

10,001 -20000ksh

20,001-40,0000

40,001-60,000ksh

60001-80000ksh

80001-100000ksh

More than 100,000ksh

**f) Harvesting**

Less than 1,000 Ksh

1,001-5,000 Ksh

5,001-10,000 Ksh

10,001 -20000ksh

20,001-40,0000

40,001-60,000ksh

60001-80000ksh

80001-100000ksh

More than 100,000ksh

**After section 1**

Section 2 of 2

Sources of farmer information

**Description (optional)**

**13a). Where do you usually get information related to maize farming practices?**

**Extension services**

Agribusiness seminars/workshops

Farmer groups

Radio/TV

Other (please specify) Option 1

**13b). How satisfied are you with the information you receive on maize farming practices?**

Very satisfied

Satisfied

Neutral

Dissatisfied

Participation in Cooperatives

**Description (optional)**

**14a). Are you a member of any agricultural cooperative?**

Yes

No

**14b). If yes, what benefits do you derive from your participation in the cooperative? (Select all that apply)**

Access to Credit

Input Procurement

Knowledge Sharing

Market Access

Training and Capacity Building

Household Income received by the household 2022-2023

Description (optional)

**15a). What is your approximate annual household income from maize farming activities?**

Less than 50,000 Ksh

50,001 - 100,000 Ksh

100,001 - 200,000 Ksh

200,001 - 500,000 Ksh

500,001 - 1,000,000 Ksh

More than 1,000,000 Ksh

Prefer not to say

**15b). What percentage of your total household income is derived from maize farming?**

Less than 10%

10% - 25%

26% - 50%

51% - 75%

More than 75%

Prefer not to say

**15 c). Do you engage in any other income-generating activities besides maize farming?**

Yes

No

**15d). If yes, please specify the other income-generating activities**

Livestock farming

Poultry farming

Dairy farming

Horticulture

Agribusiness ventures

Retail business

Employment (outside agriculture)

Handicrafts or artisan work

Transportation services

Other (please specify): ___________

**16a). How much does it cost you to transport maize to the point of sale (Ksh )**

Less than 500 Ksh

501 - 1,000 Ksh

1,001 - 2,000 Ksh

2,001 - 5,000 Ksh

More than 5,000 Ksh

I do not incur transportation costs

Prefer not to say

**16b). Where, do you take your harvested maize**

Local Market

Cooperative

National Cereal and Produce Board, (NCPB)

Milling Company

Export

On-Farm Storage

Other (please specify) ___________

I do not harvest maize Option 1

**17a). What is the distance from your homestead to the nearest Agricultural finance providers?**

Less than 5 kilometers (or 3 miles)

5-10 kilometers (or 3-6 miles)

10-20 kilometers (or 6-12 miles)

More than 20 kilometers (or 12 miles)

**17b). What is the distance from your homestead to the nearest market place for farm produce?**

Less than 5 kilometers (or 3 miles)

5-10 kilometers (or 3-6 miles)

10-20 kilometers (or 6-12 miles)

More than 20 kilometers (or 12 miles)

**17c). What is the distance from your homestead to the nearest agricultural extension service provider?**

Less than 5 kilometers (or 3 miles)

5-10 kilometers (or 3-6 miles)

10-20 kilometers (or 6-12 miles)

More than 20 kilometers (or 12 miles)

**17d). What is the type of the road from your homestead to the nearest farm produce market?**

Tarmacked Roads

Murram/Rough roads

Thank you for participating in this study!
