# Supplementary material for: The synergistic effects of microcredit access and agricultural technology adoption on maize farmer’s income in Kenya
Source: PLoS One. 2025 Jan 6;20(1):e0316014. doi: 10.1371/journal.pone.0316014 (PMC11703112; doi:10.1371/journal.pone.0316014)
Supplement: Attachment — Submitted filename: Response to Reviewers.pdf [file pone.0316014.s005.pdf]

Response to the Associate editor's comments.

Thank you for sharing your comments on our manuscript. We appreciate the critical feedback provided, which is invaluable for improving our work. To the best of our ability, we have tried to address the comment accordingly and red color track changes are evident in the manuscript. We hope the revised meets the standard for publication in Plos One.

Comment 1. "Please amend the title either on the online submission form or in your so that they are identical"

Response 1: We appreciate your constructive feedback regarding the title similarity, we have changed it to as it appears in reviewers with track changes.

The Synergistic Effects of Microcredit Access and Agricultural Technology Adoption on Maize Farmer's Income in Kenya

Reason for adjustment:

a) **Broad Geographic relevance**, all authors suggested that mentioning "Kenya" instead of a specific county (Uasin Gishu) broadens the study's appeal to an international audience. It implies that the findings may have relevance beyond the specific region.
b) **Global perspective.** A broader title might attract more readers, as specific regional names like Uasin Gishu may not resonate with those unfamiliar with the area.

Response to Reviewer #1

Comment 2: Abstract: The abstract is poorly written. The authors should start the abstract with a strong research problem statement. Then the aim of the study, materials and methods, findings of the study and conclusion and recommendation should be stated properly.

Response 2: We appreciate your constructive feedback regarding the abstract. In response, we have revised the abstract by incorporating the ideal structure and content, as suggested, and have shared the updated version with all co-authors. We have ensured that the abstract clearly begins with a strong and well-defined research problem statement, aligning with your suggestion to provide a focused and direct introduction. Following this, we have outlined the aim of the study,

the materials and methods employed, the key empirical findings, and concluded with a succinct summary of the recommendations. We believe the revised abstract now meets the expected structure and effectively summarizes the study's key elements in a concise manner, in line with your guidance as shown below:

Abstract.

Addressing global food security requires urgent improvement in agricultural productivity, particularly in developing economies where market imperfections are perverse and resource constraints prevail. While microcredit is broadly recognized as a catalyst for economic empowerment, its role in facilitating agricultural technology adoption and enhancing agricultural incomes remains underexplored. This study examines the synergistic effects of microcredit access and agricultural technology adoption on the incomes of maize farmers in Kenya. Using household-level data, we employ an endogenous switching regression framework to regulate possible endogeneity in access to microcredit. We find that microcredit access positively influences the adoption of advanced agricultural technologies. Key determinants, including marital status, use of fertilizer application, access to extension services, and cooperative membership, are found to increase the probability of microcredit access significantly. Most importantly, the Average Treatment Effect on the Treated (ATT) indicates a 40.52% income increase among farmers who access credit, attributed to the timely adoption of better-quality seeds, improved agricultural technologies, and inputs. This highlights microcredit's role in promoting allocative efficiency and enhancing Total Factor Productivity (TFP) in agricultural practices. This is attributed to the fact that robustness checks, including propensity score matching and sensitivity analyses, corroborate these findings. The study advocates for the implementation of targeted financial policies and educational initiatives to promote credit system integration, savings, and financial literacy, particularly for credit-constrained households, thereby strengthening rural financial markets and driving sustainable agricultural development across the regions.

Keywords: Microcredit, Technology Adoption, Smallholder Farmers, Endogenous Switching Regression, Total Factor Productivity, Kenya

Comment 2: The introduction explains the conceptual background and justification of a study which lead to a clear statement of research objective and scope. In introduction part, the authors describe much on microcredit aspect of different things. The authors should logically describe the issues in national and global aspect such as (1) what are the existing technologies available in case of maize farmers in Kenya? (2) what are the hinders of technology adoption? (3) what is the exiting national credit policy in the Kenya. These should be described logically.

Response 2: We appreciate your feedback regarding the abstract. In response, we shared the ideal sections of the abstract to ensure a clearer and more structured presentation with all authors as per suggestion and findings from empirical results. We have started with a stronger research problem statement which is indeed conform to your opinion as we later take keenly on importance of beginning with proper and strong straightforward statement, followed by the aim of the study, materials and methods used, key findings, and concluded with a clear summary of the recommendations. We believe the revised abstract now adheres to the expected structure and concisely summarizes the study.

2. Introduction: We appreciate your valuable feedback on the introduction. In response to your suggestions, we have made substantial revisions to this section to ensure it more clearly addresses the national and global context, as well as the rationale for the study as illustrated in paragraph 2-4 page 1 with each trying to answer the following after reading and incorporating updated data.

National and Global Context: We have restructured the introduction to logically present the issues related to maize farming in Kenya within both national and global perspectives. Specifically, we now highlight: **Existing Technologies for Maize Farmers in Kenya:** A comprehensive review of the available technologies has been included to contextualize the current practices and innovations in maize farming in Kenya.

a) Modern technologies existing in Kenya maize farmers include improved seed varieties, inorganic fertilizers, mechanization, and irrigation. However, adoption of these technologies remains low among smallholder farmers, who based on resource constraints rely on traditional practices (1). Fertilizer usage, for instance, averages 20 kg/ha far below global benchmarks in regions such as China, Brazil, India, and South Africa. This indicates a critical gap in nutrient input

that hampers crop yields (FAO, 2022). Similarly, mechanization in Kenya stands at just **25%**, implying that most farmers still depend on manual labor in various production stages which is less efficient and constrains productivity (2). Moreover, only 7% of Kenya's agricultural land is irrigated, leaving most farmers to rely on unpredictable rainfall patterns (3). This insufficient utilization of available technologies reveals the urgency of improving credit accessibility, strengthening extension services, and upgrading infrastructure to promote the adoption of modern farming technologies

b) Barriers to Technology Adoption: We have elaborated on the various hindrances that prevent farmers from adopting these technologies, including economic, social, and infrastructural challenges.

In Kenya, despite agricultural potential, smallholder farmers face several barriers to the adoption of modern farming technologies. High input costs particularly for fertilizers, improved seeds, and mechanization tools, make these technologies inaccessible to many farmers. Moreover, financial constraints remain a significant challenge, given that about 90% of rural farmers are unable to access formal credit, restricting their investment capacity (3). Additionally, inadequate rural infrastructure and weak agricultural extension services contribute to a lack of awareness and education on the benefits of new technologies. Furthermore, cultural preferences and risk aversion lead farmers to stick with traditional methods, fearing the risks associated with adopting unfamiliar practices. These factors collectively limit technological adoption in the agricultural sector.

c) National Credit Policies in Kenya: A thorough discussion of Kenya's national credit policies and their impact on farmers' access to financing for technological adoption has been added. This provides a clearer understanding of the institutional. To address financial constraints, undeniably, the Kenyan government, has introduced various credit policies and programs aimed at supporting smallholder farmers. These include the Agricultural Finance Corporation (AFC), established in 1963 to provide credit for agricultural production, and Kilimo Biashara, launched in 2008 to offer affordable loans to small-scale farmers. The Uwezo Fund, initiated in 2013, targets youth empowerment, women, and people with disabilities in agribusiness. Additionally, in a quest to reduce the unemployment crisis, the Enable Youth Kenya Program was launched in 2016 with funding from the African Development Bank (AfDB). Despite these efforts, credit access remains

constrained, especially in rural areas hindered by supply-side factors such as insufficient credit sources, unsuitable loan products, and lengthy procedures. Nevertheless, relaxation of supply-side factors, by lowering interest rates and increasing microfinance access in rural areas, have remained challenging as farmers may decline to use credit due to (i) asymmetric information, (ii) risk aversion, strict lending requirements, availability of alternative financing, (iii) cumbersome loan processes. Furthermore, non-contingency terms in credit contracts can limit microcredit demand, often leading to defaults and the seizure of household properties.

Research Gap and Rationale: We have refined the justification for the study by conducting a more rigorous review of the literature by covering all related studies particularly those relating to credit and technology adoption between 2015-2024 and this has helped us to delve deeper clarity identifying a gap in current research. This gap lies in the lack of comprehensive studies that explore the intersection of technology adoption, credit access, and agricultural development for maize farmers in Kenya. We address this gap after reading and making relevance with other existing works and finally we address as follow;

This study contributes to the literature in two folds. First, previous empirical studies have explored how access to credit can enhance technology adoption (4-8), as well as yields and productivity (9-11). However, they often focus on single technology packages and with no comprehensive consideration of income effects differentials between credit-constrained and non-credit-constrained households. Moreover, previous studies in Kenya have primarily examined the determinants of credit access technological adoption, focusing on mobile banking (12-14), identifying factors such as the occupation of the household head, access to financial training, and household credit liquidity constraints. However, there is a paucity of studies that assess the impact of credit access on technology adoption in a more unified manner, mainly concerning its effects on household income. Secondly, to the best of our knowledge, while much of the existing literature has focused on the direct effects of credit access on technology adoption, few studies have addressed the heterogeneous impacts of credit access across different agricultural yields and regionals (Girma and Research (15), Makate, Makate (16), Mariyono (17). This study aims to fill this gap by incorporating an endogenous switching regression (ESR) in examining how credit access influences technology adoption and income outcomes across different agricultural yields, considering that ESR addresses the potential existence of endogeneity and we provide insights on

policies, in augmenting and strengthen rural financial institutional partnerships within farming communities, using credit for economic empowerment, mainly by investing in technological adoption and considering flexible repayment mechanisms.

Response 3: We have revised the discussion to ensure that it is more directly linked to the results, with a clear focus on interpreting and validating our findings. Each key result is now discussed in detail, with an emphasis on how it contributes to the overall understanding of the study as shown on first line of line 2-5 on page 14-16

The results in Table 3 summarize the social economic characteristics of the maize farmers illustrating variables used in the model estimation. The dependent variable, farmers' level of technology adoption farmers using microcredit provides perceived insights into demographic and economic adoption patterns. The average age of maize farmers is 45 years, relatively younger than 46 years for non-users, indicating that farmers are relatively young, with productive capabilities of adopting technological advancement that could enhance productivity. Results corroborate empirical findings by Bakare, Ogunleye (18) and Gabriel and Gandorfer (19) who found that the age of 41-50 is a critical point of productivity and receptivity to innovation, young farmers possess the skills and risk tolerance to incorporate new agricultural technologies effectively. Additionally, findings correspond to findings by Asante-Addo, Mockshell (20) in Ghana who found that younger farmers exhibit a proactive stance toward technology adoption, distinctive attributes caused by familiarity with digital tools, and a heightened propensity for risk-taking. In contrast, as farmers age, the adoption likelihood tends to diminish, as older farmers often prioritize stability over potential yield-enhancing investments due to increased conservatism and reliance on accumulated savings rather than external financing,(21). This pattern resonates with microeconomic theories of diminishing marginal utility in risk aversion, where the marginal benefit of new technology does not adequately outweigh the perceived risks among older farmers, Sunding and Zilberman (22)

Comparison with Existing Literature: We have expanded the discussion by referencing the relevant literature, highlighting where our findings align with or differ from previous studies. This helps to contextualize our results within the broader body of research particularly on discussion part on determinants and impacts of credit access by changing the table variables particularly in

consideration of instrumented variable, we drop variable bank access as it was more correlated with other variables after testing the VIF. This has been extensively done and illustrated in last **paragraph of page 17.**

Further, both groups have adopted over 80% improved maize seed varieties, but credit users show higher adoption rates of inorganic fertilizers (89%) in comparison to noncredit users (19%) implying that access to these inputs is seemingly relatively expensive though crucial for optimal yields. Noncredit users generally have fewer than ten years of schooling, less involvement in financial institutions (52% have bank accounts versus 89% for credit users), and lower access to extension education and information. Distance to financial institutions and markets varies significantly. Credit users have shorter distances to markets (0.16 km) and financial institutions (1.98 km), whereas noncredit users face longer distances to markets (0.91 km) and financial institutions (2.00 km). Given that Table 3 does not provide differences in determinants and income impacts of credit access, a more robust approach is necessary; otherwise, an inexperienced estimator is likely to either under/or overstate the findings. Further, we tested for multicollinearity and heteroscedasticity as illustrated in Appendix Table 1A. According to Gujarati (23), VIF more than the threshold value of 10 indicates multicollinearity within covariates. Based on our results, as illustrated in Table 1A, we found (VIF) of 1.27. Additionally, results from the Breusch-Pagan (BP) test on heteroscedasticity test results in Appendix 1A show a Chi-square value of 0.025, with a p-value of 0.97. This high above p-value leads us to fail to reject the null hypothesis, confirming constant error variance and no evidence of heteroscedasticity,(24).

Country Context and Explanation of Contradictory Findings: These explanations have been integrated into the manuscript starting at the beginning of the discussion section (pages 21–24) and further elaborated on pages 27–29. We believe these revisions provide a clearer understanding of the local conditions in Kenya and offer a more robust explanation for the contradictions observed in our findings relative to the literature. We are confident that these revisions have enhanced the manuscript and addressed the concerns raised regarding the discrepancies in the results.

The average treatment effect on the treated (ATT) is estimated by calculating the average difference in income outcomes between matched treated and non-treated individuals, Rosenbaum

and Rubin (25). To ensure robustness various matching techniques were applied, including nearest neighbor matching (PSM-NNM), kernel matching, radius matching, and weighted nearest neighbor matching, following procedures by Caliendo and Kopeinig (26).

In the analysis, as shown in Table 7, the PSM-Neighbour (1) approach matched each treated individual with the five nearest non-treated individuals based on their propensity scores. In contrast, Kernel matching applied a nonparametric technique to calculate a weighted average of outcomes, with weights inversely related to the distance between treated and control observations. Radius matching was implemented with a caliper of 0.01, limiting matches to those within a specified propensity score distance. Additionally, weighted NNM, which bases similarity on a weighted function of covariates rather than propensity scores, was applied to validate the reliability of the findings, resonating with findings by Ricome, Barreiro-Hurle (27). The covariates used were consistent with those in endogenous switching regression.

The ESR model results show that access to microcredit significantly increases maize farmers' income by 40.52% compared to non-users. This finding is also consistent with the PSM results which are positive and statistically significant. However, as expected, differences are observed in Table 6 and Table 7 with PSM showing relatively higher estimates in comparison to ESR. The plausible explanation is that the ESR model controls for unobserved factors by estimating a selection equation, which can isolate treatment effects more precisely, in contrast, PSM may sometimes overestimate or underestimate treatment effects due to its reliance on observed covariates (28, 29). Nevertheless, as postulated by (30) the PSM to covariate imbalances or hidden bias, estimates of causal impacts compared to models that control for unobserved characteristics, hence these variations highlight the importance of considering unobserved heterogeneity in the analysis.

Robustness checks, including t-tests, confirm the significance of these findings at the 1% level. This implies that farmers with access to microcredit achieve higher incomes than non-credit users. This could be premised that the availability of microcredit facilitates the timely adoption of better-quality seeds, improved agricultural technologies, and inputs (Ruzzante et al., 2021). Additionally, microcredit access could be used to diversify income sources and set up insurance schemes to manage risks from climate shocks or market fluctuations (31). In contrast, other studies found that microcredit access resulted in no impact on yield improvement but it increased income on off-farm

activities (32-34), additionally (35, 36) found that in general, farmers face uncertainty or poor market access, they may prioritize consumption over long-term investments in productivity-enhancing technologies. To this end, while microcredit has shown positive effects in terms of input adoption and income growth, its overall effectiveness depends on complementary factors such as education, technical support, and risk management strategies (37). Per se, maize farmers utilized the accessed credit for the primary intended purpose, particularly the adoption of improved maize seeds and chemical fertilizers, consequently improving their income. Results corroborate the findings by Awotide, Abdoulaye (38) that access to credit improves yields and income of cassava smallholder farmers in Nigeria. Similarly, (39) in Brazil, (9), (40) in India, and additionally (41, 42) in Kenya. Both found that farmers with access to credit can easily acquire farm implements, and adopt technology and mechanized farm structures leading to an increase in total factor productivity (TFP) translating to higher income.

Comment 5: Conclusion section is nothing but repetitions of the findings. You should draw the conclusion based on findings.

Response 5: We appreciate your insightful suggestions; we have incorporated and adjust the entire conclusion as shown below:

Improving agricultural productivity through the adoption of modern technologies, particularly in food production, has garnered significant attention from global policymakers, particularly in the current rising food insecurity, increasing population pressure, and the challenges posed by climate change. Given the importance of capital as a critical factor of production, access to institutional credit, as well as informal and semi-formal financial sources like microcredit, is believed to play a key role in enabling rural farm households to adopt modern agricultural technologies. Additionally, such financial access helps mitigate consumption risks, address market imperfections through crop insurance, and ultimately drive higher productivity and technical efficiency. This study investigates the effects of microcredit access on the adoption of agricultural technology and its influence on the income of maize farmers in Kenya. A multistage sampling approach was employed to select a sample of 524 respondents. The analysis was conducted using descriptive statistics, probit regression, endogenous switching regression (ESR), and sensitivity analysis techniques to assess the collected data.

The main results from the endogenous switching regression (ESR) model reveal that marital status, household land size, availability of extension education, demand and usage of inorganic fertilizers, and cooperative membership significantly influence farmers' decisions to access credit. Furthermore, access to credit substantially maize farmers' income, with influencing factors including the source of information, land size, and availability of extension education. Most importantly, the Average Treatment Effect on the Treated (ATT) reveals that farmers with access to microcredit experience an income increase of 40.52% compared to non-users. This increase is attributed to the ease and flexibility in the acquisition of high-quality inputs, mechanizing agricultural practices, efficient use of agrochemicals, mitigating consumption risks in risk-averse households, and leverage of crop insurance. Finally, given the positive effects of microcredit from agricultural technologies as observed in the current study and other documented literature, sustainability is required upon policy reforms to relax credit constraints for rural households. Such policy adjustments would facilitate broader access to financial resources, allowing farmers to overcome credit barriers and experience further spillover effects of agricultural technology adoption. Additionally, microcredit can provide the opportunity for farmers to diversify into agricultural value chains, including processing and the cultivation of high-value crops, which are often overlooked. This diversification enables households not only to increase farm productivity but also to tap into more lucrative segments of the agricultural sector, enhancing their overall economic resilience.

Response to Editor

Methodology

Comment 1: Please ensure that you refer to Figure 1 in your text as, if accepted, production will need this reference to link the reader to the figure. We note that Figure 1 in your submission contain map/satellite images which may be copyrighted. All PLOS content is published under the Creative Commons Attribution License (CC BY 4.0), which means that the manuscript, images, and Supporting Information files will be freely available online, and any third party is permitted to access, download, copy, distribute, and use these materials in any way, even commercially, with proper attribution.

Response 1: Thank you for your excellent and insightful review. We have carefully addressed all your comments, and the revised manuscript Map as reflects these changes (red color track changes are evident). Initially, we failed to clearly indicates the source as we just indicated as survey 2024 instead of Authors thus bringing issues. We draw the map ourselves as authors and we have all the necessary references and we believe it has enhanced the manuscript requirement as shown in page 6

Response to Reviewer #2

Comment 1: The English writing and language should be improved and synergistic relationship between

Response 1: Thank you for your constructive feedback on the manuscript. We appreciate your thorough review and suggestions, which are valuable for improving the clarity and coherence of the study. To show the relationship between microcredit access**,** modern agricultural technologies**,** and agricultural income have carefully reviewed the organization of the manuscript from Table 3,5,6,7 and 8 to enhance its logical flow and clarify the interconnections as indicated in red color of review article with track changes from page 22-24 and impacts on income synergistic shown from page 27-29.

Response to Editor's Comment on Language and Clarity:

Thank you for your valuable feedback regarding the clarity and readability of our manuscript. We fully acknowledge the importance of clear and accessible communication, especially in presenting complex relationships in our study. In response to your suggestion to collaborate with a writing coach or copyeditor, we have undertaken a comprehensive process to significantly improve the manuscript's readability and coherence, as outlined below:

a) Collaboration with Linguistic Experts: After making initial revisions to the manuscript, we submitted it for review to a Professor of Linguistics at the College of International Education Foreign Affairs, Nanjing Agricultural University as suggested by our university's academic advisors. This department, which offers international education programs and in collaboration with languages department conducts language proficiency tests (e.g., TOEFL, IELTS), provided us with valuable insights on the clarity and structure of the text. Based on the feedback we received, we

addressed minor linguistic issues related to punctuation (e.g., colons and commas in the methodology section) and made small grammatical corrections, such as shown below in page 1,

Page 1. Globally, maize is most critical food source, with annual production approximately 200 million metric tons (43). In Kenya, agriculture is the backbone of the economy, contributing about 33% of the GDP and supporting 70% of the population (FAO, 2022). Maize is the primary staple food, accounting for 65% of the average caloric intake and 28% of household expenditures (FAO, 2022). Despite its significance, Kenya faces deficits in production gap, with an annual maize consumption of 5 million metric tons compared to the production capacity of only 3.5 million metric tons, resulting in reliance on imports (44). Additionally, according to (2) only 2.1 million acres out of potential arable land for maize 4.1 million acres are actively farmed, highlighting underutilization of resources (45).

To address financial constraints, undeniably, the Kenyan government, has introduced various credit policies and programs aimed at supporting smallholder farmers. These include the Agricultural Finance Corporation (AFC), established in 1963 to provide credit for agricultural production, and Kilimo Biashara, launched in 2008 to offer affordable loans to small-scale farmers. The Uwezo Fund, initiated in 2013, targets youth empowerment, women, and people with disabilities in agribusiness.

Page -14 PSM helps match credit users and non-users' groups with similar observable characteristics, thereby reducing selection bias and ensuring that comparisons between groups are valid.

Page 15 - The results in Table 3 summarize the social economic characteristics of the maize farmers illustrating variables used in the model estimation. The dependent variable, farmers' level of technology adoption farmers using microcredit provides perceived insights into demographic and economic adoption patterns.

Page 30 - Pseudo-R^2 values dropped to between 0.006 and 0.007, and likelihood-ratio tests became insignificant (p-values 0.447–0.494), revealing inefficiency differences in covariate alignments for microcredit users and non-users.

b) Further Review by Experts. After the initial linguistic revisions, we sought feedback from a group of experts, including four postdoctoral researchers with strong academic backgrounds and experience in reviewing international journal submissions. Among these experts were:

- One Chinese postdoctoral fellow (for whom English is a second language). Suggested to link causal effects on usage on conclusion section to show other possible pathways of microcredit use apart credit which we include later in revise manuscript. Most importantly, the Average Treatment Effect on the Treated (ATT) reveals that farmers with access to microcredit experience an income increase of 40.52% compared to non-users. This increase is attributed to the ease and flexibility in the acquisition of high-quality inputs, mechanization of agricultural practices, efficient use of agrochemicals, mitigating consumption risks in risk-averse households, and leverage of crop insurance.
- Two exchange Ph.D. researchers from Switzerland and the UK. They suggested on page the need to include reference in PSM and ESR discussion on response to reviewers to ease linkages. They also suggested on **Page 28 few tenses and grammar.** Robustness checks, including t-tests, confirm the significance of these findings at the 1% level. This implies that farmers with access to microcredit achieve higher incomes than non-credit users. This could be premised that the availability of microcredit facilitates the timely adoption of better-quality seeds, improved agricultural technologies, and inputs (Ruzzante et al., 2021).
- One senior counsel who serves on the editorial board of one of Chinese English review journals at our college of Economics of Management. On his suggestion Suggested that to include two factor terminologies existing in theory of production particularly when dealing with sensitive inputs such as inorganic fertilizer which it has effects and contrasting view on usage. **Page 22**.This study's findings resonate with findings (46), which relate the concept of Product-Factor Productivity. This concept proposes that the efficient allocation and use of inorganic fertilizers can significantly increase output per unit of input, thereby enhancing crop yields and stabilizing a farmer's overall income. In contrast, studies by Wu, Hao (47) found that, in certain instances, the overuse of inorganic fertilizers can affect Factor-Factor Productivity relationships with other factors of production, such as labor and capital, resulting in high upfront costs and financial risks.

c) Final Stage: Use of Online Tools: To ensure the manuscript meets the highest standards of English proficiency, we conducted a final review using online **grammar correction tools** (such as Grammarly). We focused on improving the formality of the language, eliminating slang, and ensuring that the tone was appropriate for an international audience. After addressing all suggested

grammar corrections, we carefully rechecked the document to ensure that it was free of errors. We believe that the manuscript is now in a much clearer and more polished state, and we are confident that it will be accessible and engaging for an international audience.

Final response: Thank you for your comment. We understand the importance of having a manuscript in perfect English for clarity and professionalism. We have thoroughly polished the English in the manuscript to ensure it meets the required standards. However, if the paper still does not meet the necessary linguistic standards, we are more than willing to engage a professional English proofreader to further enhance its quality.

Comment 2: Authors should supply the details output file of ESR model to check the consistency of the model. Which statistical analysis tool/software is used should be mentioned?

Response 2: We value your comments on which it enriched further our analysis.

Analytical tools/software

The data collected were then analyzed using a variety of statistical techniques, including t-tests and chi-square tests, to compare the treated and control groups across different variables. To handle missing data, listwise deletion was applied, ensuring that only complete cases were considered in the analysis. Additionally, the Endogenous Switching Regression (ESR) model was used to address potential self-selection bias and endogeneity related to the adoption of improved farming technologies. While the identified relationships were statistically significant, the effect sizes were small enough to minimizethe risk of bias due to non-randomness. All statistical analyses were performed using **STATA-18 software.**

First, throughout our analysis, we used STATA 18 software since it has robust capabilities for handling large datasets, performing advanced statistical analysis, and providing a wide range of econometric tools suited to our research objectives. Based on the analysis, we have justified the Average Treatment Effects (ATT) variations in endogenous switching regression (ESR) and Propensity Score Matching (PSM). Based on the previous results we have addressed those variations after checking more literature and similar previous peer reviewed articles from reputable journals, and several consultations from all authors and we made rectify the results as indicated in page 25–27-line paragraph 2-5. Moreover, authors also suggested we need to provide independent PSM table as to serve its robustness role and also to be clear and systematic for audience.

This consultation leads us to perform the following analysis and considerations which entails

1) **Instrument Selection and Validation:** Several consultations were held among all authors to refine the choice of instrumental variables. We selected an instrument that is highly correlated with individual adoption behavior but is uncorrelated with maize yield income, thereby satisfying the exclusion restriction necessary for valid instrumentation. This ensures that the instrument influences the outcome (maize yield income) only through its effect on adoption behavior. We believe this strengthens the causal inference in our analysis, as per the guidelines outlined by Lokshin and Sajaia (2004).

2. Addressing Multicollinearity: Upon further analysis, we identified that variables such as "bank account" and "future credit use" exhibited significant multicollinearity and lacked variability in the Kenyan context. Specifically, bank account ownership is nearly universal among adults over 18 years of age in Kenya, which rendered these variables unsuitable for inclusion in the model. As a result, we dropped these variables and focused on more relevant determinants of adoption behavior, such as access to inorganic fertilizer and improved seed, which play a critical role in maize production in Kenya.

3. **Variable Prioritization**: To ensure a more robust and theoretically sound model, we followed Analytical Hierarchical Model (AHM) principles, which allowed us to prioritize variables based on their relevance to maize production. This approach ensured that the most important determinants were emphasized in our model while minimizing the potential for noise and spurious relationships. Detailed explanations of the variable selection and prioritization process can be found on pages 8–11 of the manuscript. For further explanations and variations, we also sought other references cited within this text as to show variations of PSM and ESR in impact evaluation process as shown in reference section.

4. Finally, Quality Matching in Propensity **Score Matching (PSM)** with robust methods, we identified persistent imbalance in some covariates. To address this, we implemented alternative matching algorithms, including **kernel matching**, **nearest neighbor matching**, and **optimal matching**, as illustrated in **Table 8**. These adjustments helped to improve covariate balance, identify the sources of variation, and confirming that the matching process effectively minimized selection bias, enhancing the reliability of the impact estimates.

Response to Plausible Explanation for Divergences and variations in PSM and ESR

The ESR model's ability to control for unobserved heterogeneity through a selection equation leads to a more precise estimation of treatment effects. The ESR model estimates a two-outcome

equation corresponding to adopters and non-adopters (conditional on their selection criterion) in a two-stage framework**,** (3) This is reflected in the ATT estimates derived from ESR, which are slightly lower than those of PSM. Specifically, the ESR model accounts for unobserved factors influencing both the decision to access microcredit and income generation, making its estimates more robust. By contrast, the PSM approach relies solely on observable covariates for matching treated and untreated households. This reliance on observable variables can lead to overestimation or underestimation of treatment effects due to omitted variable bias Caliendo andKopeinig (4) For instance, in **Table 7,** PSM using kernel and nearest-neighbor matching methods produces ATT estimates of 51.29% and 49.82%, respectively, which are higher than the ESR estimate of 40.52%. This divergence highlights the potential for PSM to inflate treatment effects due to its **failure to account for unobserved heterogeneity**. Similar findings have been reported in other studies, where PSM has been observed to overstate the benefits of interventions due to its reliance on balanced matching and the quality of covariates used for matching. The plausible explanation is that the ESR model controls for unobserved factors by estimating a selection equation, which can isolate treatment effects more precisely, in contrast, PSM may sometimes **overestimate or underestimate treatment effects**, (5, 6) due to its reliance on observed covariates (7-10). Nevertheless, as postulated by (11) the PSM to covariate imbalances or hidden bias, estimates of causal impacts compared to models that control for unobserved characteristics, hence these variations highlight the importance of considering unobserved heterogeneity in the analysis.

Table 6: Impacts of microcredit participation on household income from the ESR model

Adoption status	N	**Income from credit**	**Non-credit users**	**Treatment effect**	**% Change**
Adopters	368	12.31	8.76	ATT:3.55*** (0.026)	40.52
Non-adopters	156	11.68	8.45	ATU: 3.23***(0.050)	38.22

Note: Statistical significance at the 1% level. Standard errors are shown in parentheses. Credit access impact on technology adoption. ATT – Average Treatment Effect on the Treated; ATU – Average Treatment effects on untreated.

Source: Authors compilation

Table 7: Robustness checks; Propensity score matching (PSM) estimates impacts on income

Matching type	**Income from credit**	**Non-credit users**	**Treatment effect ATT**	**% Change**
Nearest neighbor (1)	12.29	8.19	4.08	49.82
Kernel	12.29	8.11	4.16	51.29
Radius	12.29	8.45	3.82	45.44

Notes: *** Statistical significance at the 1% level; ATT – Average Treatment Effect on the Treated.

Quality of balancing

Table 8 presents the result for the quality of the matching using several key indicators to ensure adequate covariate balance observed in credit users and non-users. Before matching, the Pseudo R^2 was estimated at 0.033, indicating a modest explanatory power of the model. The likelihood-ratio test further confirmed the presence of significant differences in the distribution of covariates between the two groups ($p < 0.001$, indicating substantial differences in covariate distributions between the two groups suggesting that the model had strong explanatory power in distinguishing treated from control observations. The mean standardized bias was also relatively high at 8.9%, reflecting considerable imbalance. After implementing the matching procedure, the covariate balance improved significantly across all methods. Pseudo-R^2 values dropped to between 0.006 and 0.007, and likelihood-ratio tests became insignificant (p-values 0.447–0.494), revealing inefficiency differences in covariate alignments for microcredit users and non-users. The standardized mean difference, or Mean bias, was reduced by approximately half to 4.4%–4.8%, below the 5% threshold for adequate balance,(27). These results confirm that the matching process effectively minimized selection bias, enhancing the reliability of the impact estimates.

	Pseudo R2	p>chi2	Mean Bias	Pseudo R2	p>chi2	Mean Bias	Pseudo R2	p>chi2	Mean Bias
Unmatched	0.033	0.000	8.9	0.033	0.000	8.9	0.033	0.000	8.9

Matched	0.007	0.405	5.1	0.007	0.447	4.4	0.007	0.447	4.6

Table 8: Quality of matching

PSM-NNM **Kernel** **Radius**

Comment on Stata PDF output.

Response: we have provided the **Stata output file** along with the corresponding commands as attached at end of this reviewer response page.

References

1. Kubitza C, Krishna VV. Instrumental variables and the claim of causality: Evidence from impact studies in maize systems. Global Food Security. 2020;26:100383.
2. Lokshin M, Sajaia Z. Maximum likelihood estimation of endogenous switching regression models. The Stata Journal. 2004;4(3):282-9.
3. Amadu FO, McNamara PE, Miller DC. Yield effects of climate-smart agriculture aid investment in southern Malawi. Food Policy. 2020; 92:101869.
4. Caliendo M, Kopeinig S. Some practical guidance for the implementation of propensity score matching. Journal of economic surveys. 2008;22(1):31-72.
5. Zhao Z. Using Matching to Estimate Treatment Effects: Data Requirements, Matching Metrics, and Monte Carlo Evidence. The Review of Economics and Statistics. 2004;86(1):91-107.
6. Chen JW, Maldonado DR, Kowalski BL, Miecznikowski KB, Kyin C, Gornbein JA, et al. Best Practice Guidelines for Propensity Score Methods in Medical Research: Consideration on Theory, Implementation, and Reporting. A Review. Arthroscopy: The Journal of Arthroscopic & Related Surgery. 2022;38(2):632-42.
7. Ouya FO, Murage AW, Pittchar JO, Chidawanyika F, Pickett JA, Khan ZR. Impacts of climate-resilient push–pull technology on farmers' income in selected counties in Kenya and Tanzania: propensity score matching approach. Agriculture & Food Security. 2023;12(1):15.
8. Stuart EA. Matching methods for causal inference: A review and a look forward. Stat Sci.

2010;25(1):1-21.

9. 14. Tesfay MG. Does fertilizer adoption enhance smallholders' commercialization? An endogenous switching regression model from northern Ethiopia. Agriculture & Food Security. 2020;9:1-18.

10. 15. Balana B, Oyeyemi M. Credit constraints and agricultural technology adoption: Evidence from Nigeria: Intl Food Policy Res Inst; 2020.

11. 16. Rayhan SJ, Rahman MS, Lyu K. The role of rural credit in agricultural technology adoption: The case of Boro rice farming in Bangladesh. Agriculture. 2023;13(12):2179.

12. 17. Yigezu YA, Mugera A, El-Shater T, Aw-Hassan A, Piggin C, Haddad A, et al. Enhancing adoption of agricultural technologies requiring high initial investment among smallholders. Technological Forecasting and Social Change. 2018;134:199-206.

13. 18. Giné X, Yang D. Insurance, credit, and technology adoption: Field experimental evidencefrom Malawi. Journal of development Economics. 2009;89(1):1-11.

14. 19. Abate GT, Rashid S, Borzaga C, Getnet K. Rural finance and agricultural technology adoption in Ethiopia: does the institutional design of lending organizations matter? World Development. 2016;84:235-53.

15. 20. Jimi NA, Nikolov PV, Malek MA, Kumbhakar S. The effects of access to credit on productivity: separating technological changes from changes in technical efficiency. Journal of Productivity Analysis. 2019;52(1):37-55.

16. 21. Diamoutene AK, Jatoe JBD. Access to credit and maize productivity in Mali. Agricultural Finance Review. 2021;81(3):458-77.

17. 22. Assouto AB, Houngbeme DJ-L. Access to credit and agricultural productivity: Evidence from maize producers in Benin. Cogent Economics & Finance. 2023;11(1):2196856.

18. 23. Schroeder E. The impact of microcredit borrowing on household consumption in Bangladesh. Applied Economics. 2020;52(43):4765-79.

19. 24. Ouattara NB, Xueping X, Bi TBAY, Traoré L, Ahiakpa J, Olounlade OA. Determinants of smallholder farmers' access to microfinance credits: A case study in Sassandra-Marahoué District, Côte d'Ivoire. Agricultural Finance Review. 2020;80(3):401-19.

20. 25. Eslava M, Freixas X. Public development banks and credit market imperfections. Journal of Money, Credit and Banking. 2021;53(5):1121-49.

21. 26. Aydin D. Consumption response to credit expansions: Evidence from experimental assignment of 45,307 credit lines. American Economic Review. 2022;112(1):1-40.

22. 27. Aldieri L, Brahmi M, Chen X, Vinci CP. Knowledge spillovers and technical efficiency for cleaner production: An economic analysis from agriculture innovation. Journal of Cleaner Production. 2021;320:128830.

23. 28. Liu X, Zhao Q. Banking competition, credit financing and the efficiency of corporate technology innovation. International Review of Financial Analysis. 2024;94:103248.

24. 29. Kiplimo JC, Ngenoh E, Koech W, Bett JKJJoD, Economics A. Determinants of access to credit financial services by smallholder farmers in Kenya. 2015;7(9):303-13.

25. 30. Ogada MJ, Mwabu G, Muchai DJA, economics f. Farm technology adoption in Kenya: a simultaneous estimation of inorganic fertilizer and improved maize variety adoption decisions. 2014;2(1):1-18.

26. 31. Njogu GK, Olweny T, Njeru A. Relationship between farm production capacity and agricultural credit access from commercial banks. 2018.

```
  ___  ____  ____  ____  ____(R)
 /__    /   ____/   /   ____/
___/   /   /___/   /   /___/
  Statistics/Data analysis

    User: Plos One Review
Project: Final revision for reviewer
```

```
1 . movestay(Lincome = Age gender Maritak_status Education_level Livestock_stock tlandsize Extension_education Occu
  > nd_ownership Nonfarm_income socialinteractions  experience cooperative_membership sourcesof_information ),selec
  > _finainstitute)
```

```
Fitting initial values .....
Iteration 0:  Log likelihood = -734.23009
Iteration 1:  Log likelihood = -733.50294
Iteration 2:  Log likelihood = -733.49534
Iteration 3:  Log likelihood = -733.49534

Endogenous switching regression model           Number of obs   =        524
                                                Wald chi2(15)   =      68.65
Log likelihood = -733.49534                     Prob > chi2     =     0.0000
```

	Coefficient	Std. err.	z	P>|z|	[95% conf.	interval]
**Lincome_1**						
Age	.0009118	.0038407	0.24	0.812	-.0066159	.0084395
gender	-.1475438	.0902108	-1.64	0.102	-.3243537	.0292661
Maritak_status	.1474849	.0984925	1.50	0.134	-.0455569	.3405267
Education_level	.0392316	.0926224	0.42	0.672	-.142305	.2207683
Livestock_stock	.2006166	.0892143	2.25	0.025	.0257598	.3754734
tlandsize	.1301335	.0378607	3.44	0.001	.0559279	.2043391
Extension_education	.3759638	.1177821	3.19	0.001	.1451151	.6068125
Occupation_household	.0942329	.0900749	1.05	0.295	-.0823107	.2707764
ferti_dummy	.4037827	.1697917	2.38	0.017	.070997	.7365683
land_ownership	-.0320823	.0183333	-1.75	0.080	-.0680149	.0038502
Nonfarm_income	-.2574939	.3241801	-0.79	0.427	-.8928752	.3778873
socialinteractions	-.0710297	.1694208	-0.42	0.675	-.4030884	.261029
experience	-.1179532	.1021948	-1.15	0.248	-.3182513	.082345
cooperative_membership	.3746484	.125616	2.98	0.003	.1284455	.6208513
sourcesof_information	-.3721678	.1178903	-3.16	0.002	-.6032285	-.1411071
_cons	11.95272	.8318744	14.37	0.000	10.32228	13.58317
**Lincome_0**						
Age	.0039111	.0056587	0.69	0.489	-.0071797	.0150019
gender	-.0045232	.1610774	-0.03	0.978	-.3202291	.3111827
Maritak_status	.3482988	.1678955	2.07	0.038	.0192297	.6773679
Education_level	.3702404	.1758142	2.11	0.035	.025651	.7148298
Livestock_stock	-.0672427	.1445849	-0.47	0.642	-.3506239	.2161386
tlandsize	.0730483	.0551578	1.32	0.185	-.035059	.1811556
Extension_education	.5408758	.1630428	3.32	0.001	.2213177	.8604338
Occupation_household	.2341746	.1454373	1.61	0.107	-.0508772	.5192264
ferti_dummy	.0841208	.3007908	0.28	0.780	-.5054183	.6736599
land_ownership	.0028599	.053689	0.05	0.958	-.1023685	.1080884
Nonfarm_income	-.1625118	.3098881	-0.52	0.600	-.7698814	.4448578
socialinteractions	.045633	.2135461	0.21	0.831	-.3729097	.4641756
experience	.0024115	.1484296	0.02	0.987	-.2885052	.2933281
cooperative_membership	-.4468251	.1809613	-2.47	0.014	-.8015028	-.0921473
sourcesof_information	-.3078545	.151007	-2.04	0.041	-.6038228	-.0118861
_cons	8.1594	.8330288	9.79	0.000	6.526694	9.792107
**Access_microcredit**						
Age	.0026326	.0082036	0.32	0.748	-.0134461	.0187113
gender	-.1272943	.2032523	-0.63	0.531	-.5256614	.2710728
Maritak_status	.497287	.2060014	2.41	0.016	.0935318	.9010423
Education_level	.1335993	.1965944	0.68	0.497	-.2517187	.5189173
Livestock_stock	.1448685	.1899492	0.76	0.446	-.227425	.5171621
tlandsize	.0730458	.0772814	0.95	0.345	-.0784229	.2245145

```
  Occupation_household |  -.2061775    .191229    -1.08   0.281    -.5809795    .1686244
           ferti_dummy |   1.707701   .1934368     8.83   0.000     1.328571     2.08683
        land_ownership |   .0488141   .0488259     1.00   0.317    -.0468828     .144511
    socialinteractions |  -.0219694   .2936849    -0.07   0.940    -.5975813    .5536424
            experience |   .1874676   .2100845     0.89   0.372    -.2242904    .5992255
cooperative_membership |   .5425764   .2179523     2.49   0.013     .1153979     .969755
  sourcesof_information |  .1523035   .2161928     0.70   0.481    -.2714266    .5760336
   Extension_education |   .5826743   .2194962     2.65   0.008     .1524697    1.012879
        Nonfarm_income |   .4078119   .5266318     0.77   0.439    -.6243674    1.439991
Distance_finainstitute |    1.33219   .2018422     6.60   0.000     .9365862    1.727793
                 _cons |  -5.071221   1.363214    -3.72   0.000    -7.743071    -2.39937
-----------------------------------------------------------------------------------------
         /lns1 |  -.2554207   .0387663    -6.59   0.000    -.3314011   -.1794402
         /lns2 |  -.2179833   .0678092    -3.21   0.001    -.3508868   -.0850797
           /r1 |   .2714854   .2325392     1.17   0.243     -.184283    .7272539
           /r2 |   .4758125   .3089177     1.54   0.123    -.1296551     1.08128
---------------+------------------------------------------------------------------
       sigma_1 |   .7745906    .030028                       .7179171    .8357379
       sigma_2 |   .8041389    .054528                       .7040635    .9184391
         rho_1 |   .2650065   .2162083                      -.1822249    .6213824
         rho_2 |   .4428837   .2483247                      -.1289335    .7936733
----------------------------------------------------------------------------------
LR test of indep. eqns. :          chi2(1) =     3.75   Prob > chi2 = 0.0529
----------------------------------------------------------------------------------

  2 .
    end of do-file

  3 . mspredict ATTreal,yc1_1

  4 .
  5 . mspredict ATUreal,yc2_2

  6 .
  7 . mspredict ATTcounter,yc2_1

  8 .
  9 . mspredict ATUcounter,yc1_2

 10 .
 11 . ttest ATTreal = ATTcounter , unpaired

Two-sample t test with equal variances
------------------------------------------------------------------------------
Variable |     Obs        Mean    Std. err.   Std. dev.   [95% conf. interval]
---------+--------------------------------------------------------------------
 ATTreal |     368    12.31793    .0176372    .3383394    12.28325    12.35261
ATTcou~r |     368    8.762894    .0193171    .3705657    8.724908     8.80088
---------+--------------------------------------------------------------------
Combined |     736    10.54041    .0668547    1.813724    10.40916    10.67166
---------+--------------------------------------------------------------------
    diff |            3.555036    .0261576                3.503683    3.606389
------------------------------------------------------------------------------
    diff = mean(ATTreal) - mean(ATTcounter)                       t = 135.9085
H0: diff = 0                                     Degrees of freedom =      734

    Ha: diff < 0                 Ha: diff != 0                 Ha: diff > 0
 Pr(T < t) = 1.0000         Pr(|T| > |t|) = 0.0000          Pr(T > t) = 0.0000
```

```
12 .
13 . ttest ATUcounter = ATUreal , unpaired

   Two-sample t test with equal variances
```

Variable	Obs	Mean	Std. err.	Std. dev.	[95% conf.	interval]
ATUcou~r	156	11.68684	.0322591	.4029164	11.62312	11.75057
ATUreal	156	8.454752	.0390112	.4872497	8.37769	8.531814
Combined	312	10.0708	.095058	1.679059	9.88376	10.25784
diff		3.232093	.0506214		3.132488	3.331698

```
    diff = mean(ATUcounter) - mean(ATUreal)                      t =  63.8484
H0: diff = 0                                     Degrees of freedom =      310

    Ha: diff < 0                 Ha: diff != 0                 Ha: diff > 0
 Pr(T < t) = 1.0000         Pr(|T| > |t|) = 0.0000          Pr(T > t) = 0.0000

14 . do "C:\Users\SHADRA~1\AppData\Local\Temp\STD3b34_000000.tmp"

15 . psmatch2 Access_microcredit Age gender Maritak_status Education_level Livestock_stock  Extension_education Occu
   > and_ownership Nonfarm_income socialinteractions experience cooperative_membership Distance_finainstitute source
   >  com n(1)

   Probit regression                                       Number of obs =    524
                                                           LR chi2(15)   = 384.25
                                                           Prob > chi2   = 0.0000
   Log likelihood = -126.94293                             Pseudo R2     = 0.6021
```

Access_microcredit	Coefficient	Std. err.	z	P>|z|	[95% conf.	interval]
Age	.0030456	.008129	0.37	0.708	-.0128869	.0189781
gender	-.1186938	.2025304	-0.59	0.558	-.5156461	.2782585
Maritak_status	.5304515	.2032559	2.61	0.009	.1320773	.9288256
Education_level	.1721209	.1970505	0.87	0.382	-.214091	.5583328
Livestock_stock	.1350112	.1872202	0.72	0.471	-.2319336	.501956
Extension_education	.5746741	.2140225	2.69	0.007	.1551978	.9941505
Occupation_household	-.1673096	.1898619	-0.88	0.378	-.5394322	.2048129
ferti_dummy	1.740372	.1907539	9.12	0.000	1.366501	2.114242
land_ownership	.0547193	.0488444	1.12	0.263	-.0410139	.1504525
Nonfarm_income	.3684066	.526193	0.70	0.484	-.6629128	1.399726
socialinteractions	.0370049	.2944143	0.13	0.900	-.5400366	.6140464
experience	.1885707	.2119404	0.89	0.374	-.226825	.6039663
cooperative_membership	.5790298	.2172411	2.67	0.008	.1532451	1.004815
Distance_finainstitute	1.260618	.19938	6.32	0.000	.8698403	1.651395
sourcesof_information	.1880036	.2152626	0.87	0.382	-.2339033	.6099105
_cons	-4.988074	1.348669	-3.70	0.000	-7.631416	-2.344731

Variable	Sample	Treated	Controls	Difference	S.E.	T-stat
Lincome	Unmatched	12.3177637	8.45693047	3.86083318	.082623787	46.73
	ATT	12.2827039	8.19959606	4.08310786	.26370487	15.48

Note: S.E. does not take into account that the propensity score is estimated.

psmatch2: Treatment assignment	psmatch2: Common support Off suppo	On suppor	Total
Untreated	0	156	156
Treated	100	268	368
Total	100	424	524

```
16 .
   end of do-file

17 . do "C:\Users\SHADRA~1\AppData\Local\Temp\STD3b34_000000.tmp"

18 . psmatch2 Access_microcredit Age gender Maritak_status Education_level Livestock_stock  Extension_education Occu
   > and_ownership Nonfarm_income socialinteractions experience cooperative_membership Distance_finainstitute source
   >  com kernel
```

Probit regression   Number of obs =   524
                                                         LR chi2(15)   = 384.25
                                                         Prob > chi2   = 0.0000
Log likelihood = -126.94293   Pseudo R2   = 0.6021

Access_microcredit	Coefficient	Std. err.	z	P>|z|	[95% conf.	interval]
Age	.0030456	.008129	0.37	0.708	-.0128869	.0189781
gender	-.1186938	.2025304	-0.59	0.558	-.5156461	.2782585
Maritak_status	.5304515	.2032559	2.61	0.009	.1320773	.9288256
Education_level	.1721209	.1970505	0.87	0.382	-.214091	.5583328
Livestock_stock	.1350112	.1872202	0.72	0.471	-.2319336	.501956
Extension_education	.5746741	.2140225	2.69	0.007	.1551978	.9941505
Occupation_household	-.1673096	.1898619	-0.88	0.378	-.5394322	.2048129
ferti_dummy	1.740372	.1907539	9.12	0.000	1.366501	2.114242
land_ownership	.0547193	.0488444	1.12	0.263	-.0410139	.1504525
Nonfarm_income	.3684066	.526193	0.70	0.484	-.6629128	1.399726
socialinteractions	.0370049	.2944143	0.13	0.900	-.5400366	.6140464
experience	.1885707	.2119404	0.89	0.374	-.226825	.6039663
cooperative_membership	.5790298	.2172411	2.67	0.008	.1532451	1.004815
Distance_finainstitute	1.260618	.19938	6.32	0.000	.8698403	1.651395
sourcesof_information	.1880036	.2152626	0.87	0.382	-.2339033	.6099105
_cons	-4.988074	1.348669	-3.70	0.000	-7.631416	-2.344731

Variable	Sample	Treated	Controls	Difference	S.E.	T-stat
Lincome	Unmatched	12.3177637	8.45693047	3.86083318	.082623787	46.73
	ATT	12.2827039	8.11310376	4.16960016	.214507903	19.44

Note: S.E. does not take into account that the propensity score is estimated.

psmatch2: Treatment assignment	psmatch2: Common support Off suppo	On suppor	Total
Untreated	0	156	156
Treated	100	268	368
Total	100	424	524

```
19 .
   end of do-file

20 . do "C:\Users\SHADRA~1\AppData\Local\Temp\STD3b34_000000.tmp"

21 . psmatch2 Access_microcredit Age gender Maritak_status Education_level Livestock_stock  Extension_education Occu
   > and_ownership Nonfarm_income socialinteractions experience cooperative_membership Distance_finainstitute source
   >  com radius
```

Probit regression

Number of obs =	**524**
LR chi2(**15**) =	**384.25**
Prob > chi2 =	**0.0000**
Pseudo R2 =	**0.6021**

Log likelihood = **-126.94293**

Access_microcredit	Coefficient	Std. err.	z	P>|z|	[95% conf.	interval]
Age	**.0030456**	**.008129**	**0.37**	**0.708**	**-.0128869**	**.0189781**
gender	**-.1186938**	**.2025304**	**-0.59**	**0.558**	**-.5156461**	**.2782585**
Maritak_status	**.5304515**	**.2032559**	**2.61**	**0.009**	**.1320773**	**.9288256**
Education_level	**.1721209**	**.1970505**	**0.87**	**0.382**	**-.214091**	**.5583328**
Livestock_stock	**.1350112**	**.1872202**	**0.72**	**0.471**	**-.2319336**	**.501956**
Extension_education	**.5746741**	**.2140225**	**2.69**	**0.007**	**.1551978**	**.9941505**
Occupation_household	**-.1673096**	**.1898619**	**-0.88**	**0.378**	**-.5394322**	**.2048129**
ferti_dummy	**1.740372**	**.1907539**	**9.12**	**0.000**	**1.366501**	**2.114242**
land_ownership	**.0547193**	**.0488444**	**1.12**	**0.263**	**-.0410139**	**.1504525**
Nonfarm_income	**.3684066**	**.526193**	**0.70**	**0.484**	**-.6629128**	**1.399726**
socialinteractions	**.0370049**	**.2944143**	**0.13**	**0.900**	**-.5400366**	**.6140464**
experience	**.1885707**	**.2119404**	**0.89**	**0.374**	**-.226825**	**.6039663**
cooperative_membership	**.5790298**	**.2172411**	**2.67**	**0.008**	**.1532451**	**1.004815**
Distance_finainstitute	**1.260618**	**.19938**	**6.32**	**0.000**	**.8698403**	**1.651395**
sourcesof_information	**.1880036**	**.2152626**	**0.87**	**0.382**	**-.2339033**	**.6099105**
_cons	**-4.988074**	**1.348669**	**-3.70**	**0.000**	**-7.631416**	**-2.344731**

Variable	Sample	Treated	Controls	Difference	S.E.	T-stat
Lincome	Unmatched	**12.3177637**	**8.45693047**	**3.86083318**	**.082623787**	**46.73**
	ATT	**12.2827039**	**8.45693047**	**3.82577345**	**.057627449**	**66.39**

Note: S.E. does not take into account that the propensity score is estimated.

psmatch2: Treatment assignment	psmatch2: Common support		
	Off suppo	On suppor	Total
Untreated	**0**	**156**	**156**
Treated	**100**	**268**	**368**
Total	**100**	**424**	**524**

```
22 .
   end of do-file

23 .
```
