# Supplementary material for: The synergistic effects of microcredit access and agricultural technology adoption on maize farmer’s income in Kenya
Source: PLoS One. 2025 Jan 6;20(1):e0316014. doi: 10.1371/journal.pone.0316014 (PMC11703112; doi:10.1371/journal.pone.0316014)
Supplement: Attachment — Submitted filename: Review_report_Plos_one 2.docx [file pone.0316014.s004.docx]

Review Report

**to the**

**“PLOS ONE”**

**On**

“**The Synergistic Effects of Microcredit Access and Agricultural Technology Adoption on Maize Farmer's Income in Uasin Gishu County, Kenya**”

This study examines the “Synergistic Effects of Microcredit Access and Agricultural Technology Adoption on Maize Farmer's Income in Kenya”. This is a good piece of work indeed. Therefore it is crucial to investigate the availability and existing credit scenario in the Kenya. Definitely microcredit is crucial to the small and medium enterprises for adoption of any technology. Besides these some others factors are related in case of technology adoption. However, I have made some comments about the following aspects that need to be addressed before accept by the journal.

1. **Abstract**: The abstract is poorly written. The authors should start the abstract with a strong research problem statement. Then the aim of the study, materials and methods, findings of the study and conclusion and recommendation should be stated properly.
2. **Introduction:** The introduction explains the conceptual background and justification of a study which lead to a clear statement of research objective and scope. In introduction part, the authors describe much on microcredit aspect of different things. The authors should logically describe the issues in national and global aspect such as (1) what are the existing technologies available in case of maize farmers in Kenya? (2) what are the hinders of technology adoption? (3) what are the exiting national credit policy in the Kenya. These should be described logically. I hereby suggest the authors to highlight the issue on country context. Therefore, country context should be stated in terms of global scholarship in a logical way. Unfortunately, the authors have failed to demonstrate the rationale of the study. Therefore, the authors are hereby advised to find a research gap by a rigorous review of literature.

3. **Methodology:** The lower part of the study area is nothing but repetition of justification.

The study area section should contain why the location is selected for study such as climate and weather condition, geographical position, intensive growing area of maize. Why the location is important for selected crop as well as technological advancement. The PSM technique should be stated in analytical part as you used it for robustness check of the model.

4. **Results and discussion:**

The discussion part should result oriented and should be validate with the existing finding of the review of literature and contradict determinants should be explained with the country context. The ATT value of ESR model is much vary from PSM model with NNM and Kernel matching method. However, number of significant determinants is also low which require further investigation. **Therefore, I want see the output pdf file with command to check the inconsistencies.**

5. **Conclusion:** Conclusion section is nothing but repetitions of the findings. You should draw the conclusion based on findings.

Finally, the authors should have to keep in mind that the readers of this journal are from all over the world. As an international article it should be readable. The authors should response the above comments before it accepted for publication in this esteemed journal.
